# Transcriptome profiling of two contrasting pigeon pea (*Cajanus cajan*) genotypes in response to waterlogging stress

**DOI:** 10.3389/fgene.2022.1048476

**Published:** 2023-01-10

**Authors:** Anshika Tyagi, Sandhya Sharma, Harsha Srivastava, Anuradha Singh, Tanvi Kaila, Sajad Ali, Ambika B. Gaikwad, N. K Singh, Kishor Gaikwad

**Affiliations:** ^1^ National Institute for Plant Biotechnology, New Delhi, India; ^2^ Department of Biotechnology Yeungnam University, Gyeongsan, South Korea; ^3^ ICAR-National Bureau of Plant Genetic Resources, New Delhi, India

**Keywords:** pigeon pea, waterlogging stress, RNAseq, BUSCO, functional annotation, signaling

## Introduction

Pigeon pea (*Cajanus cajan* L.) is one of the important legume crop that contributes significantly to the nutritional stability and economy of billions of people in most developing nations (Sharma et al., 2020). Like other legumes, pigeon pea often offers more balanced and nutrient-dense calories and proteins (20%–22%) than cereals, making them essential in terms of food security. It is the sixth most significant legume food crop in the world, with a cultivation area of about 5 million hectares (ha) (Varshney et al., 2012). However, environmental stressors pose a persistent threat to the pigeon pea crop’s production, yield, and quality. Among them waterlogging is one of the most harmful stress in pigeon pea which results in huge yield and economic losses around the world (Sultana et al., 2013; Tyagi et al., 2017; Tyagi et al., 2022). Overall, waterlogging has been observed to cause an annual loss of about .28–1.1 million tons per hectare which reduces production by 25%–30% in pigeon pea (Sultana, 2010; ICRISAT, 2011; Bansal and Srivastava, 2012). Waterlogging affects pigeon pea at all growth stages, but is more severe during the seedling and vegetative phases, thereby showing wilting, senescence and chlorosis (Bansal and Srivastava, 2012). Additionally, waterlogging also makes pigeon pea plants more susceptible to fungal diseases like Fusarium wilt and Phytophthora blight which results in significant yield losses (Yohan et al., 2017). Generally, pigeon pea is grown in low-input, risk-prone marginal environments and low-lying places that are more susceptible to waterlogging (Varshney et al., 2012; Duhan and Sheokand 2020). During waterlogging, the inhibition of aerobic respiration hinders growth and a variety of developmental processes, including seed germination, vegetative growth, and subsequent reproductive growth (Pan et al., 2021). Additionally, in waterlogged soils, ethylene and carbon dioxide levels increase dramatically in the root area which in turn alters the functions of soil microbiome that leads to an intense de-nitrification and accumulation of ammonium and polyphenolic compounds (Arduini et al., 2019). Also, it restricts the availability of nutrients like nitrogen (N) and sulphur (S) or changes them into a form that plants cannot absorb. It also changes ion homeostasis zinc (Zn), phosphorous (P), manganese (Mn), and iron (Fe), which can reach lethal levels to plants (Arduini et al., 2019). The most common effect of waterlogging stress is oxygen deficiency (hypoxia) and ethylene accumulation in plants, which can restrict root growth and root permeability, both of which lead to cell death (Sasidharan et al., 2018; Pan et al., 2021). However, plants use their multifaceted defense system in response to waterlogging stress by regulating their morphological, biochemical and molecular traits. For example, the formation of aerenchyma in roots is one of the main traits in plants that confer waterlogging tolerance (Luan et al., 2018). At the physiological and biochemical levels, plants produce numerous molecules such as osmolytes, calcium (Ca^2+^), reactive oxygen species (ROS), hormones, antioxidants that confers waterlogging tolerance (Pan et al., 2021). However, the molecular traits that confers waterlogging tolerance is least understood with many knowledge gaps. For example, how plants perceive waterlogging stress and triggers signal transduction pathways that in turn leads the expression of stress responsive genes. Additionally, the role of different sensors or receptors, ion channels, nicotinamide adenine dinucleotide phosphate (NADPH) etc that are involved in waterlogging signal transduction warrants future investigation.

Unlike other crops, pigeon pea genetic advancement has been hampered by scarce genomic resources and a lack of genetic variety in the basic gene pool which pose a significant obstacle to its improvement in terms of stress resistance and yield (Bohra et al., 2010; Varshney et al., 2012). Although there has been significant advancement in understanding the complexity of waterlogging signaling dynamics in model and cereal plants (Eysholdt-Derzsó et al., 2017; Yamauchi et al., 2018), but there is limited information in the most of the legume crops particularly in pigeon pea. For instance, the defense signaling pathways, hormonal crosstalk, regulatory genes, and transcriptional factors involved in waterlogging tolerance in pigeon pea cultivars remains enigmatic despite the availability of high throughput tools. Previous studies have identified many sensitive and tolerant pigeon pea genotypes which were mainly based on the morphological, physiological, biochemical traits and days of survival (Sultana et al., 2013). Although different pigeon pea waterlogging genotypes were found by these investigations, it is still largely unknown how these genotypes control waterlogging tolerance at the molecular level. Therefore, it is necessary to decode the waterlogging tolerance in pigeon pea cultivars and identify potential target genes for developing future climatic smart resilient pigeon pea genotypes in order to maintain productive agriculture and ensuring food security. In this work we first studied the effect of waterlogging stress in two contrasting pigeon pea genotypes viz.*,* JBP-110B (tolerant) and ICP 7035 (sensitive) as well as their transcriptional profiling using *De-novo* transcriptome assembly (unpublished data). This comprehensive transcriptomic study data has led the important findings on the differentially expressed genes regulating waterlogging signaling mechanism in susceptible and tolerant pigeon pea genotypes which can be applied to subsequent research on the improvement of waterlogging resilience in pigeon pea and other legume crops.

### Value of the data

• Pigeon pea is a rich source of protein for poor vegetarian people widely grown in Indian, Africa and Southeast Asia subcontinent.
• Waterlogging is the most detrimental abiotic stress in pigeon pea. Despite the availability of high through put tools, genomic resource for waterlogging tolerance trait in pigeon pea remains unknown.
• In this study, we have generated a comprehensive global gene expression profiling dataset for two contrasting pigeon pea genotypes, JBP-110B (tolerant) and ICP 7035 (sensitive) using *De novo* RNA-seq analysis. A total of 39.2 GB of RNA seq data were confirmed by Benchmarking universal Single-Copy Orthologs (BUSCO) and gene ontology (GO) enrichment analysis using Illumina Hiseq paired-end sequencing.
• This transcriptomic data can provide novel insights in waterlogging signaling mechanism and also aids in the identification of potential target genes, transcriptional factors and other key molecular players.

## Material and methods

### Plant material and waterlogging stress treatment

Seeds of two contrasting pigeon pea genotypes JBB-110B (Tolerant) and ICP 7035 (Sensitive) were sown in pots (0.8 m deep and 12 m diameter) containing autoclaved soilrite mixture in three biological replicates under controlled lighting and temperature conditions, with a maximum temperature of 30°C–32°C and a minimum temperature of 22°C–25°C in the Phenomics Facility (PF) at National Institute for Plant Biotechnology (NIPB), New Delhi. For both genotypes, 5 seeds were initially planted in each pot. After 14 days of germination, the plants were then thinned to three healthy plants and allowed to grow for 4 weeks. One-month old plants were exposed to waterlogging stress for 7 days. Briefly, pots were dipped in a plastic tray containing water and water level was maintained at 5 cm level above the soil. Leaf tissue samples from control and waterlogging stress conditions were collected (at the fourth day/first visible waterlogging induced symptom) in three biological replicates, immediately dip into liquid nitrogen and finally stored at −80°C for further processing.

### RNA extraction, transcriptome library preparation, and sequencing

Total RNA was isolated from each sample (control and waterlogging treated) as per the protocol mentioned in Spectrum Plant Total RNA Extraction Kit (SIGMA). Eight RNA samples were taken for library preparation in duplicates. Overall, eight transcriptome libraries were prepared. The RNA samples were quantified using Nanodrop and Qubit. The input concentration of RNA was taken as 1 µg for library preparation. QIAseq^®^ Stranded mRNA Select kit (Qiagen) was used for transcriptome library preparation. PolyA mRNA was enriched from total RNA, and then it was fragmented followed by first strand synthesis. Then second strand synthesis, end repair and A-addition were done. The adapters were ligated and the library was amplified by polymerase chain reaction (PCR). The final library was quantified by qubit and quality check was done using Agilent Bioanalyzer (Agilent 2,100). With the help of Agilent DNA High Sensitivity kit, the library size distribution was assessed, and the library was run on Illumina Hiseq platform.

### Quality control and *de novo* transcriptome assembly

Initial quality control (QC) of both the samples in biological replicates was carried out using FastQC version v0.11.9 to check the per base sequence quality of the raw reads of leaf transcriptome. Adapter removal and trimming was performed by TrimGalore version v0.6.1 (https://www.bioinformatics.babraham.ac.uk/projects/trim_galore/) and FASTX-Toolkit version v0.0.14 (http://hannonlab.cshl.edu/fastx_toolkit), respectively. Thereafter, the results of all samples were run on MultiQC version v1.12 (Ewels et al., 2016). After filtering, the *de novo* assembly was performed in the Trinity software version v2.14.0 with default parameters (Grabherr et al., 2011). We further used BUSCO tool version v4 to evaluate the overall completeness of the final transcriptome assembly (Manni et al., 2021). From an evolutionary perspective, it is fair to predict that these genes will be found in a given genome as single copies, hence BUSCO is excellent for determining assembly completeness. For this work, we utilized the transcriptome evaluation mode with the eukaryote lineage database (eukaryota orthoDB9). The CD-HIT software version v4.6.1 was used to obtain non-redundant unigenes (Li and Godzik, 2006; Fu et al., 2012). OmicsBox version v2.1 (https://www.biobam.com/omicsbox/) was employed for annotation based on the GO terms *viz.* Cellular components, molecular functions, and biological processes. Finally, KEGG (Kyoto encyclopedia of genes and genomes) pathway enrichment analysis of all the DEGs was carried out using OmicsBox (Götz et al., 2008).

## Results

### RNA-seq and *de novo* transcriptome assembly

**FIGURE 1:**
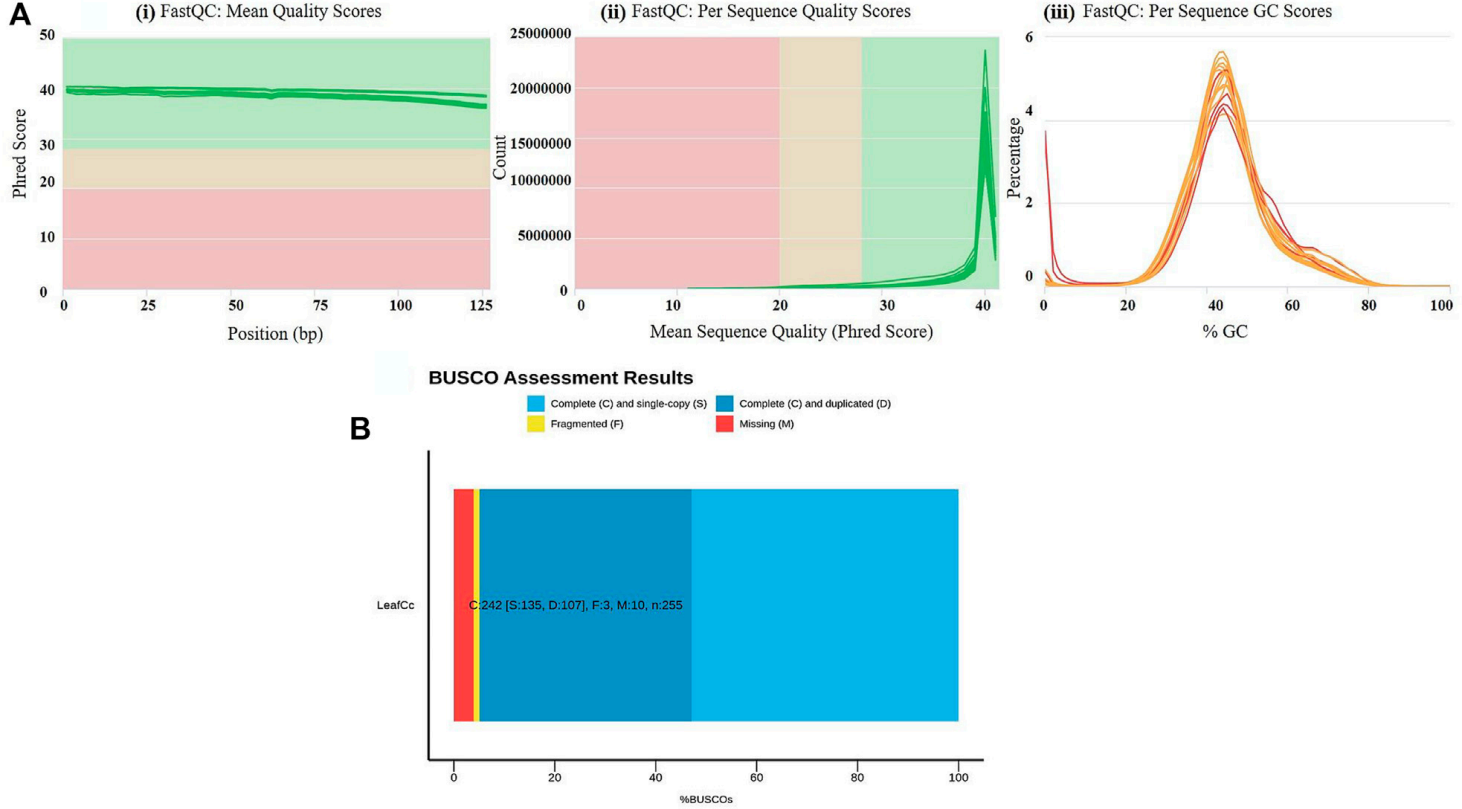
**(A)** Results of raw read preprocessing. (i) Mean quality scores per read. The *x*-axis represents the mean quality scores, and the *y*-axis depicts the read counts. (ii) Per sequence quality scores. The *x*-axis represents the position, and the *y*-axis depicts the Phred score. (iii) GC content of reads. The *x*-axis represents the GC content, and the *y*-axis depicts the ratio of reads. Quality assessment metrics for trimmed and filtered RNA-Seq data used to make the *de novo* transcriptome assembly. **(B)** % BUSCO assessment results of leaf RNAseq data in *C. cajan* for quality check and completeness analysis showing maximum number of unigenes categorized in complete (C), single copy (S), and duplicated (D) genes.

**FIGURE 2:**
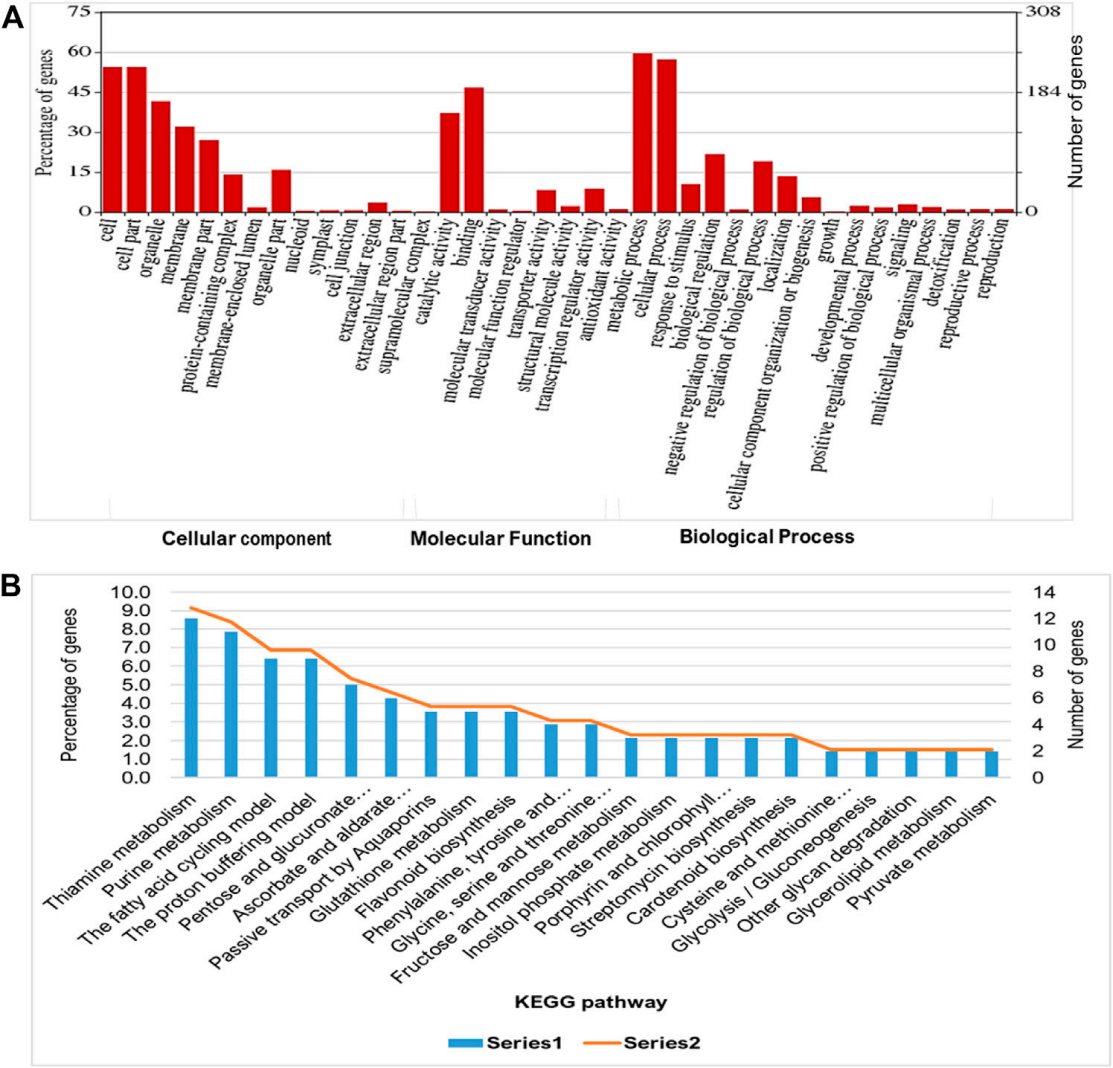
Functional enrichment analysis of predicted transcript targets during waterlogging stress in pigeon pea: **(A)** Gene ontology (Cellular components, molecular function, and biological process) and **(B)** KEGG pathway analysis to annotate the unigenes for waterlogging RNAseq data using BLASTX program.

To evaluate the waterlogging tolerance or susceptibility, we first screened numerous pigeon pea genotypes based on morphological, physiological, and biochemical parameters (unpublished data). Firstly, we checked the quality control (QC) of both the samples using FastQC version v0.11.9 to examine the per base sequence quality of the raw reads of leaf transcriptome (Figure 1A). Based on the findings, we chose two distinct pigeon pea genotypes-JBP-110B (tolerant) and ICP 7035 (sensitive)-for RNA sequencing. Illumina Hiseq sequencing run produced a total of 217,090,406 and 262,705,712 raw paired-end reads of 150 × 2 bp (base pair) from 8 RNA libraries in JBP-110 (tolerant) and ICP 7035 (sensitive) genotypes. A total of 211,324,886 clean reads from the JBP-110 and 255,693,868 from the ICP 7035 genotypes were obtained after the removal of poly-A tails, adapters, primer, short and low-quality sequences using trimming process. Additionally, Trinity software was used for the *de novo* assembly of the pooled reads (467,018,754) from both samples. There were 1,457,155 transcripts in total, with an average length of 545.10 bp and a N50 value of 1984 bp. Our assembly was shown to be relatively complete by BUSCO analysis, with 94.9% (*n* = 242) of BUSCOs being full sequences, just 1.2% (*n* = 3) being fragmented sequences, and 3.9% (*n* = 10) being absent in the assembly with eukaryotic lineage (Figure 1B). Using CD-HIT software, a total of 90,084 unigenes with an average length of 1,411.77 bp and a N50 value of 2,229 bp were obtained after *de novo* assembly. The unigenes had an average guanine-cytosine (GC) content of 42.62%. The *de novo* assembly statistics summary of the RNA seq data is shown in Table 1. Annotation results showed that, in total, 66,106 (73.3%) unigenes annotated from non-redundant (NR), GO and KEGG databases. Among them the maximum number of hits related to transcriptional regulation, integral component of membrane, metal ion binding, and thiamine metabolism were found to be dominant (Figures 2A, B). The dataset generated from all the samples used in current transcriptome analysis (BioProject: PRJNA637701) are deposited in the National Center for Biotechnology Information (NCBI) Sequence Read Archive (SRA) database with accession number (SRR11940026, SRR11940027, SRR11940028, SRR11940029). This data can also be utilized for comparative studies with data from other crops to identify similarities and differences in their adaptive responses to waterlogging.

**TABLE 1:** Statistical summary of RNA-seq data used in this study.

Assembly	Trinity_cdhit
contigs ( ≥ 0 bp)	90084
contigs ( ≥ 1,000 bp)	45590
contigs ( ≥ 5,000 bp)	1549
contigs ( ≥ 10000 bp)	312
contigs ( ≥ 25000 bp)	47
contigs ( ≥ 50000 bp)	5
Total length ( ≥ 0 bp)	127177704
Total length ( ≥ 1,000 bp)	104368225
Total length ( ≥ 5,000 bp)	13665753
Total length ( ≥ 10000 bp)	5832885
Total length ( ≥ 25000 bp)	1637564
Total length ( ≥ 50000 bp)	294277
contigs	65036
Largest contig	62035
Total length	118440920
GC (%)	42.62
N50	2229
N75	1456
L50	16524
L75	32872
N’s per 100 kbp	0

## Conclusion

In the years between 2006 and 2016, floods were responsible for about two-thirds of all crop loss and destruction globally, amounting to huge yield losses (Food and Agriculture Organization of the United Nations, 2017). Similarly, waterlogging has been a major concern in legume crops especially in pigeon pea which requires timely improvement in order to maintain crop productivity. Pigeon pea an orphan crop has been neglected for its trait improvement despite being an important crop for under developed countries. In this context, we systematically studied the effect of waterlogging stress in two contrasting pigeon pea genotypes and their transcriptional profiling. To date, this is the first comparative dataset for *De-novo* transcriptome profiling under waterlogging stress in pigeon pea. The candidate unigenes discovered in this study will be extremely important for additional thorough research, such as single nucleotide polymorphisms (SNP) calling and novel non-coding RNAs such as microRNAs (miRNAs), and long non-coding RNAs (lncRNAs), aside from studies of differential gene expression, to elucidate the molecular mechanisms governing waterlogging tolerance in pigeon pea. Additionally, using gene editing or overexpression, we might modulate their expression and functionally validate them to develop waterlogging tolerant pigeon pea cultivars.

## Data Availability

The datasets presented in this study can be found in online repositories (SRA database, http://www.ncbi.nlm.nih.gov/sra) with the following accession information: BioProject ID: PRJNA637701. The names of the repository and accession number can be found in the article.
